# Does objectively measured physical activity modify the association between early weight gain and fat mass in young adulthood?

**DOI:** 10.1186/s12889-017-4924-1

**Published:** 2017-11-25

**Authors:** Elin Kolle, Bernardo L. Horta, Jonathan Wells, Soren Brage, Fernando C. Barros, Ulf Ekelund, Pedro C. Hallal

**Affiliations:** 1 Department of Sports Medicine, Norwegian School of Sport Sciences, P.O. Box 4014, Ullevål Stadion, N-0806 Oslo, Norway; 2 Postgraduate Program in Epidemiology, Federal University of Pelotas, Pelotas, Brazil; 3 Childhood Nutrition Research Centre, UCL Institute of Child Health, London, UK; 4 Medical Research Council Epidemiology Unit, University of Cambridge, Cambridge, UK; 5 Catholic University of Pelotas, Pelotas, Brazil

**Keywords:** Physical activity, Conditional weight gain, Adiposity, Young adulthood

## Abstract

**Background:**

Substantial evidence suggests that weight gain in early life is associated with increased adiposity and other metabolic disorders later in life. It is, however, unknown whether physical activity (PA) may modify these associations. We aimed to examine whether objectively measured PA at 30 years modified the associations between conditional weight gain in infancy (0–2 y) and childhood (2–4 y) with fat mass index (FMI) and visceral abdominal fat measured at age 30 years.

**Methods:**

Prospective birth cohort study in Pelotas, Brazil, including 1874 participants with weight data at birth, two and four years of age, and measures of FMI, visceral abdominal fat and PA at a mean age of 30.2 years. At age 30, time spent (min/day) in moderate-to-vigorous physical activity (MVPA) was measured objectively using a wrist-worn accelerometer worn for four to seven consecutive days.. Multiple linear regression analyses was performed to assess the associations between conditional weight gain and outcome variables at 30 years, adjusting for covariates. We examined whether PA modified the association between conditional weight gain and the outcomes of interest by introducing an interaction term (conditional weight gain × PA) in the models.

**Results:**

Conditional weight gain in infancy and childhood were both positively associated with later FMI (infancy weight gain: β = 0.68, 95% CI: 0.48, 0.88; *P* < 0.001; childhood weight gain: β = 0.91, 95% CI: 0.70, 1.11; *P* < 0.001). A formal test for interaction suggested that MVPA at 30 years of age modified the association between childhood relative weight gain and later FMI (β = −0.006, 95% CI: -0.011, −0.001; *P* = 0.029), suggesting stronger associations between weight gain and FMI in those with lower levels of MVPA. Conditional weight gain in childhood was also positively associated with visceral abdominal fat (β = 0.24, 95% CI: 0.15, 0424, *P* < 0.001). There was no evidence for a modification of the latter association after adjustment for physical activity.

**Conclusion:**

Conditional weight gain between 2 and 4 years of age is associated with increased FMI at age 30 years. However, higher levels of MVPA appear to attenuate this detrimental association.

## Background

There is compelling evidence suggesting that low birth weight is associated with metabolic disturbances such as hypertension, dyslipidemia, overweight and obesity, type 2 diabetes and cardiovascular disease risk later in life [1–4]. More recently, studies have also found rapid postnatal growth to be associated with increased risk of these disorders. In high income countries, positive associations have been reported between rapid growth during infancy and early childhood and adult obesity [5] and visceral adipose tissue [6]. In low- and middle-income countries, weight trajectories in the first two years of life has been found to be more strongly related to adult fat-free mass than to fat mass, while weight trajectories in early childhood has been found to predict both fat mass and fat-free mass [7]. Further, using data from the 1982 Pelotas birth cohort study, it was observed that rapid growth particularly after two (for women) and four (for men) years of age was associated with increased visceral fat at 23 years of age [8].

**Fig. 1:**
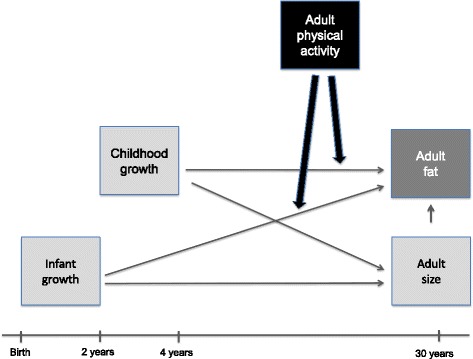
Schematic diagram to illustrate how physical activity may modify the association between infancy and childhood growth and adiposity at 30 years of age

Moreover, substantial evidence shows that regular physical activity (PA) during the life span is inversely associated with adiposity and metabolic disturbances [9–11]. It could therefore be hypothesized that higher levels of PA in adulthood may be beneficial in attenuating the association between rapid weight gain early in life and adiposity later in life (Fig. 1) [12]. Few studies have examined whether objectively measured PA can modify the association between birth weight and metabolic risk factors in adolescence or adulthood [13, 14], and the results are inconclusive. In European adolescents, Ridgway et al. [13] found no modification of accelerometer measured physical activity on the association between low birth weight and adiposity or insulin resistance. Conversely, Ortega et al. [14] reported that physical activity measured by accelerometers modified the association between birth weight and insulin resistance. However, neither of these studies examined whether physical activity modified the association between conditional weight gain and later health outcomes.n. We are not aware of any prospective studies examining if PA in adulthood can moderate or modify the associations between accurately measured infant and childhood growth on detailed measures of adult adiposity.

The aim of the present study was therefore to examine whether objectively measured PA in young adulthood modifies the associations between conditional weight gain in infancy (0–2 y) and early childhood (2–4 y) and fat mass index (FMI) and visceral abdominal fat measured at 30 years of age.

## Methods

### Study design and participants

Pelotas is a 340,000 inhabitant city in southern Brazil, close to the border of Uruguay. In 1982, 99.2% of all live born infants whose families lived in the urban area of Pelotas participated in a longitudinal birth cohort study (*n* = 5914). To date, the cohort has had nine follow-up visits. At the mean ages of 2, 4 and 30 years, we aimed to recruit all cohort participants. Follow-up rates at these ages were 87.2, 84.1, and 68.1%, respectively. The analyses presented in this paper are based on participants in these three waves of data collection. More detailed information on the study methodology has been described elsewhere [15–17]. The study was approved by the Ethics Committee of the Medicine School of the Federal University of Pelotas. Written informed consent from the participants or their parents or caretakers was obtained prior to each wave of data collection.

### Measurements

Birth weight was measured at the hospital using regularly calibrated pediatric scales (Filizolla, Brazil) to the nearest 0.1 kg. Birth length was not recorded. In the follow-up visits at ages 2 and 4 years, height and weight were measured at the participant’s home. At age 2 and 4 years anthropometric measurements were collected while subjects were in their underwear and without shoes. Weight was measured using an electronic scale (SECA-UNICEF) to the nearest 0.1 kg and height was measured using a stadiometer (CMS) to the nearest 0.1 cm.

The latest follow-up took place at the research clinic when the participants were approximately 30 years old (mean age 30.2 years). Body volume was evaluated by air displacement plethysmography, using the BodPod® (Cosmed, Italy) while the participant was wearing a tight-fitting swimming costume and a swimming cap [18]. Body volume was obtained after adjustments for predicted thoracic lung volume and estimated surface area artifact. Fat mass (FM) was calculated by software provided by the manufacturer according to the Siri eq. (21). These data were expressed relative to height: fat mass index (FMI) = fat mass (kg) / height (m)^2^. We performed abdominal ultrasound examination using a Toshiba Xario (Toshiba Medical Systems Corp) ultrasound machine with a 3.5-MHz convex probe following validated protocols [19]. Briefly, visceral fat thickness was estimated by the distance between the peritoneum and the lumbar spine at the intersection between the xyphoid line and the waist circumference. The distance was measured from static images taken at the end of a quiet expiration when applying minimal pressure. The relative intra-observer technical error of measurement for the visceral thickness was 4.1%, whereas the relative inter-observer technical error of measurement was 3.1% [19].

At the 30 year follow-up, PA was measured objectively using a wrist-worn accelerometer (GENEActiv, Kimbolton, Cambs, United Kingdom). The participants wore the accelerometer 24 h for four to seven consecutive days, following the visit at the research clinic. The GENEActiv is a triaxial accelerometry-based activity monitor, and the monitors were initialized to collect data at 87.5 Hz. Data were uploaded using the GENEActiv PC software version 2.2. Accelerometer data in binary format were analyzed with R-package GGIR (https://cran.r-project.org/web/packages/GGIR/index.html) [20]. We used mean daily minutes spent in moderate-to-vigorous physical activity (MVPA) per day as a measure of PA. MVPA was defined as an intensity >100 m*g*, based on 5 s epoch and 10 min bout durations where more than 80% of data points had to be equal to or above the threshold. The intensity threshold is based on a recent validation study [21] and has been used previously [22]. Details on the accelerometer data collection, processing and handling are available elsewhere [22]. Participants were included in the analyses if providing at least two days of accelerometer data.

### Statistical analyses

Weight gain at a particular age correlate with size at earlier ages, as well as regression to the mean, and we therefore used conditional growth modelling [23]. At each measurement point, conditional relative weight was calculated as the residual from linear regression of present weight accounting for present height and all previous weight and height measures. For instance, weight-for-age z-score at 2 years was predicted by birth weight for gestational z-score and length-for-age z-score at 2 years, and the standardized residual between actual and predicted weight z-score was calculated for each individual. The conditional variables represent children’s deviation from the expected size on the basis of their previous measures and the growth of the other children in the cohort, and can be interpreted as representing faster or slower relative weight gain [24]. Conditional size was expressed as z-scores using the internal distribution.

Our analyses initially compared the cohort members with complete data sets with those excluded from the analyses, using χ^2^ tests or independent samples t-tests. We performed multiple linear regression analyses to assess the associations between conditional weight gain (0–2 y and 2–4 y) and outcome variables at 30 years (FMI and visceral abdominal fat). Our model was adjusted for sex, birth weight for gestational age, family income at birth, maternal schooling at birth, pre-pregnancy maternal body mass index, maternal smoking in pregnancy, and breastfeeding. This model was then repeated, adjusting for minutes of MVPA to investigate if this variable moderated the association between conditional weight gain and FMI and visceral abdominal fat. We also examined whether PA modified the association between conditional weight gain and the outcomes of interest by introducing an interaction term; conditional weight gain × physical activity (MVPA, min/day as a continuous variable) in the models. Finally, if a significant interaction term was observed we repeated the regression analyses stratified by tertiles of conditional weight gain. All analyses were performed using IBM SPSS Statistics 21 (IBM Corporation, Route, Somers, NY, USA).

## Results

A total of 3701 individuals participated in the follow-up at age 30 years. Data on height and weight measures needed for computing conditional weight variables (birth weight for gestational age, weight and height at age 2 and 4 years) were available in 2512 participants. Valid accelerometer data were available in 2718 participants, FMI data were available in 3521 participants and visceral abdominal fat data were available in 3493 participants. All individuals with complete data on conditional weight gain, PA, and one of the outcome measures were included in the analyses (*N* = 1874).

Cohort participants included in the analyses were similar to those who were excluded in terms of most variables of interest. However, there was a lower prevalence of low birth weight infants (*P* = 0.001), a lower percentage of mothers with no schooling at birth (*P* = 0.008), and a lower percentage of participants coming from a family with a low income (*P* = 0.001) among those who were included in the analyses (Table 1).

**Table 1:** Characteristics of the cohort members included in the analyses (*N* = 1874) compared to the remaining participants (*N* = 1790) in terms of sociodemographic characteristics and outcomes measured at age 30 years

Variable	Included	Excluded	*P*-value
% males	48.6	49.1	0.77
% low birth weight (<2500 g)	5.8	8.6	0.001
% obese mothers pre-pregnancy (≥30 kg/m^2^)	5.1	4.1	0.32
% mothers with no schooling	3.9	5.8	0.008
% mothers smoking during pregnancy	33.8	36.1	0.13
Family income (%)			
≤ 1	18.2	21.5	0.001
1.1–3	51.7	46.6	
3.1–6	20.1	19.2	
> 6	10.0	12.7	
Body Mass Index (kg/m^2^)	26.9 (5.6)	26.7 (5.5)	0.24
Fat mass index (FMkg/m^2^)	8.8 (4.6)	8.6 (4.5)	0.06
Visceral abdominal fat (cm)	5.9 (2.1)	5.9 (2.1)	0.55

Values are percentage or mean and SD

Infancy conditional weight gain (0–2 y) was positively associated with FMI at 30 years (β = 0.68, 95% confidence interval (CI): 0.48, 0.88; *P* < 0.001) (Table 2). Adjustment for covariates and MVPA slightly attenuated the association (β = 0.62, 95% CI: 0.43, 0.82; *P* < 0.001). A formal test for interaction showed no evidence that the association between infancy conditional weight gain and FMI was modified by time spent in MVPA (β = 0.002, 95% CI: -0.004, 0.008; *P* = 0.52).

**Table 2:** Crude and adjusted associations between infancy (0–2 y) and childhood (2–4 y) conditional weight gain and fat mass index (FMkg/m^2^) and visceral abdominal fat (cm) at 30 years of age

	Crude		Model 1		Model 2	
	Β coefficient (95% CI)	*P*-value	Β coefficient (95% CI)	*P*-value	Β coefficient (95% CI)	*P*-value
Fat mass index (FMkg/m^2^)						
0–2 y	0.68 (0.48, 0.88)	<0.001	0.65 (0.45, 0.84)	<0.001	0.62 (0.43, 0.82)	<0.001
2–4 y	0.91 (0.70, 1.11)	<0.001	0.98 (0.78, 1.18)	<0.001	1.03 (0.83, 1.22)	<0.001*
Visceral abdominal fat (cm)						
0–2 y	0.07 (−0.02, 0.17)	0.14	0.05 (−0.05, 0.14)	0.34	0.04 (−0.05, 0.13)	0.42
2–4 y	0.24 (0.15, 0.34)	<0.001	0.14 (0.05, 0.23)	0.003	0.15 (0.06, 0.24)	0.001

Model 1: Adjusted for child sex, birth weight for gestational age, family income at birth, maternal schooling at birth, maternal body mass index pre-pregnancy, maternal smoking in pregnancy, and breastfeeding

Model 2: Adjusted for model 1 + moderate-to-vigorous physical activity

*Significant interaction between conditional weight gain and moderate-to-vigorous physical activity, P = 0.029

**Fig. 2:**
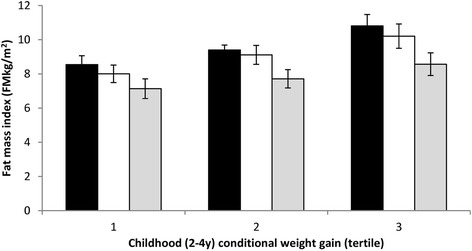
Associations between conditional weight gain (2–4 y) and fat mass index (FMkg/m^2^), stratified by tertiles of moderate-to-vigorous physical activity (MVPA, minutes per day). Data (means, 95% CI) are adjusted for child sex, birth weight for gestational age, family income at birth, maternal schooling at birth, maternal body mass index pre-pregnancy, maternal smoking in pregnancy, and breastfeeding. Black bars, low MVPA; white bars, moderate MVPA; grey bars, high MVPA. *P* = 0.029 for interaction between conditional weight gain and MVPA

Childhood conditional weight gain (2–4 y) was also positively associated with FMI at 30 years (β = 0.91, 95% CI: 0.70, 1.11; *P* < 0.001) (Table 2). Following adjustment for covariates and time spent in MVPA the association was somewhat strengthened (β = 1.03, 95% CI: 0.83, 1.22; *P* < 0.001). A formal test for interaction suggested that MVPA modified the association between childhood relative weight gain and FMI (β = −0.006, 95% CI: -0.011, −0.001; *P* = 0.029). The magnitude of the association was weaker in the lowest tertile of conditional weight gain (β = 0.73, 95% CI: 0.13, 1.33; *P* = 0.018) compared with the middle (β = 1.62, 95% CI: 0.18, 3.07; *P* = 0.028) and highest tertile (β = 1.22, 95% CI: 0.69, 1.74; *P* < 0.001). Figure 2 show FMI stratified by tertiles of conditional weight gain and MVPA (min/day). In each tertile of conditional weight gain, higher levels of MVPA was associated with substantially lower FMI and this association was most pronounced in the highest tertile of conditional weight gain.

Infancy conditional weight gain was not associated with visceral abdominal fat (β = 0.07, 95% CI: -0.02, 0.17; *P* = 0.14), and this association remained unchanged after further adjustment for covariates and time spent in MVPA per day (Table 2). In opposite, childhood conditional weight gain was positively associated with visceral abdominal fat (β = 0.24, 95% CI: 0.15, 0.34; *P* < 0.001). Adjustment for covariates and MVPA attenuated the association between childhood conditional weight gain and visceral abdominal fat (β = 0.15, 95% CI: 0.06, 0.24; *P* < 0.001). However, a formal test for interaction showed no evidence that the association between childhood weight gain and visceral abdominal fat was modified by levels of MVPA in young adulthood (β = −0.001, 95% CI: -0.003, 0.002; *P* = 0.53). We did, however, find an inverse cross-sectional association between time spent in MVPA and visceral abdominal fat at 30 years of age when adjusting for covariates (*P* = 0.001).

## Discussion

In this large birth cohort, we observed that infant conditional weight gain was positively associated with increased FMI at 30 years, whereas childhood conditional weight gain was positively associated with both increased FMI and visceral fat at 30 years. Time spent in MVPA at 30 years did not moderate the association between infant weight gain and FMI, or the association between childhood conditional weight gain and visceral abdominal fat. However, time spent in MVPA modified the association between childhood relative weight gain and FMI, suggesting stronger associations between weight gain and FMI in those with lower levels of MVPA.

Our results extend those from a few previous studies that have used objective measures of physical activity when examining whether physical activity levels modifies the relationship between birth weight and later metabolic risk factors [13, 14]. Whilst a study from the European Youth Heart Study did not find that time spent in MVPA modified the association between low birth weight and adiposity or insulin resistance in 9- and 15-year old adolescents [13], mean PA level (expressed as mean counts/min) modified the association between birth weight and insulin resistance in another group of European 14–15 year olds [14]. In the latter study, the modifying effect was observed following adjustment for current body mass index, which may indicate that the association is caused by change in size between the time points rather than early programming [12, 25]. We examined the influence of MVPA on the association between conditional weight gain and adiposity in 30 year olds, whereas the two previous studies were performed in 14–15 year old adolescents. Also, we examined conditional weight gain in infancy and childhood with later adiposity whereas the other studies examined the association between birth weight and later metabolic health. This is important as low birth weight infants usually compensate by showing a rapid growth during the first year of life, and it has been suggested that it is this postnatal adaptation in growth, rather than low birth weight itself, that contributes more to later disease risks.

Conditional weight gain early in life appears to be associated with several measures of adiposity later in life [6–8, 23]. Conditional weight gain in infancy and during childhood were associated with later FMI whereas conditional weight gain in childhood was also associated with abdominal fat mass. Rapid weight gain from birth was associated with FMI at 30 years, whereas rapid weight gain from two years was associated with both increased FMI and visceral abdominal fat. However, the association between conditional weight gain with FMI was greater in magnitude for childhood than for infancy weight gain. This suggests that rapid weight gain from two years have more effect on fat and less effect on height compared to rapid weight gain earlier in life. Our findings support the hypothesis that higher levels of MVPA may attenuate the detrimental effect of conditional weight gain in childhood on FMI in young adulthood. Obesity is a major worldwide public health problem, and some of the major health risks associated with obesity are cardiovascular disease, type 2 diabetes, and cancer of colon and breast [26, 27]. Prevention of obesity is therefore an important public health target. Our analyses shows that in all tertiles of weight gain in childhood, higher MVPA was associated with lower FMI. Additionally, in the highest tertile of conditional weight gain, the group with most MVPA (third tertile) had similar levels of FMI as the lowest tertile of MVPA in the lowest tertile of childhood conditional weight gain. This observation may have important public health implications as it may be possible to prevent the detrimental effect of high conditional weight gain by being physically active.

Despite the finding that levels of MVPA at 30 years modified the association between conditional weight gain and FMI, we did not find this modifying effect of PA on the association between conditional weight gain in childhood and visceral abdominal fat. The ability to detect a modification effect of PA on the association between conditional weight gain in early life and health outcomes later in life depends on several factors, including the precision of the measurement of the exposure and the outcome. Although the ultrasound method used to measure visceral abdominal fat is considered good, we cannot rule out that the potential effect of measurement error or variability in the measure affect our ability to detect a modification effect of PA. We did, however, find an inverse cross-sectional association between time spent in MVPA and visceral abdominal fat at 30 years. This suggests that increased time in MVPA in young adulthood might reduce visceral abdominal fat. However, we cannot rule out a bi-directional association due to the cross-sectional analysis. The accumulation of visceral abdominal fat has been particularly associated with increased risk of several diseases, such as type 2 diabetes and cardiovascular disease [28]. Therefore, we will emphasize the need of effective strategies to promote optimum linear growth particularly after the age of 2 years as well as strategies aiming to increase the PA level of all age groups.

There are several strengths with this study. This is a cohort study with data obtained at birth, during infancy and childhood with follow-up data at age 30 years. The follow-up rate at 30 years was 68.1%, which is similar to what has been reported in similar studies [17]. Also, the sample size is large and we have included measurement of several potential confounders. Further, we used state-of-the-art methods to measure the adiposity outcomes at 30 years. We also included objective measures of PA at the 30 years follow-up, which is more precise than self-reported measures of PA [29]. Finally, the use of the conditional weight gain eliminates the correlation between growth variables in subsequent age ranges. This approach is intended to remove the confounding by earlier height and weight measurements and shed light on the role of weight gain at different ages.

Some limitations need to be considered. Firstly, many participants had missing values for gestational age, and were consequently excluded from the analyses. Gestational age was self-reported, and was lacking to a greater extent in women with no or low education. Thus, our analytical sample included a lower prevalence of mothers with no schooling. Secondly, our results are restricted to subjects with complete information on growth measurements at birth and age two and four years. Further measurements at age one were available only for a small sub-sample, therefore these data have not been included in our analyses. Previous data from the Pelotas birth cohorts have shown that weight gain between birth and six months has less effect on later adiposity than weight gain in the second year of life [30], but we were not able to disentangle this effect. Thirdly, the outcome variables (FMI and visceral abdominal fat) were measured at the same time point as the effect modifier (MVPA) with no intermediate measures of MVPA, which limits our ability to draw causal relationships.

## Conclusion

In conclusion, time spent in MVPA did not modify the association between infancy weight gain and later FMI or the association between childhood conditional weight gain and visceral abdominal fat. In contrast, MVPA modified the association between childhood conditional weight gain and FMI suggesting a more pronounced association in those with high childhood weight gain.

## Abbreviations

FMI: Fat mass index
MVPA: Moderate to vigorous physical activity
PA: Physical activity
